# Dupilumab induces hair regrowth in pediatric alopecia areata: a real-world, single-center observational study

**DOI:** 10.1007/s00403-024-03225-4

**Published:** 2024-07-23

**Authors:** Eden David, Neda Shokrian, Ester Del Duca, Marguerite Meariman, Jacob Glickman, Sabrina Ghalili, Seungyeon Jung, Kathryn Tan, Benjamin Ungar, Emma Guttman-Yassky

**Affiliations:** 1 Department of Dermatology, and Laboratory of Inflammatory Skin Diseases, Icahn School of Medicine at Mount Sinai, 5 East 98th Street, New York, NY 10029 USA; 2 Albert Einstein College of Medicine, New York, NY USA; 3 School of Medicine, Vanderbilt University, Nashville, TN USA

**Keywords:** Alopecia areata, Dupilumab, Atopy, Pediatric, Adverse events, Real-world data

## Abstract

Alopecia areata (AA) is nonscarring hair loss characterized by Th1 and concomitant Th2 skewing, particularly in atopic patients. Despite novel developments for adult AA, safe and effective treatments for pediatric patients remain limited. Dupilumab, with a well-studied safety profile, may have therapeutic potential for atopic pediatric AA. To evaluate the ability of dupilumab to regrow hair in pediatric AA patients. We conducted a single-center, retrospective, observational study to evaluate hair regrowth [using Severity of Alopecia Tool (SALT)] with dupilumab in 20 children with both AD and AA (age range 5–16 years, mean 10.8 years; baseline SALT range 3–100, mean 54.4). Patient demographics, atopic history, IgE and SALT scores were collected at 12wk follow-up visits, up to > 72wks, to evaluate hair regrowth. Spearman correlations with clinical data were performed. Patients showed clinical improvement over the follow-up period (range 24 to > 72wks, mean 67.6wks) with significant mean(± SD) reduction in SALT at 48wks versus baseline [20.4(± 35.1) vs 54.4(± 37.6), respectively; p < 0.01] and continued improvement up to > 72wks [2.2(± 4.9), p < 0.01]. Baseline SALT positively correlated with disease duration (r = 0.54, p < 0.01), and negatively correlated with improvement in SALT at weeks 24, 36, and 48 (|r|≥ 0.65, p < 0.01 for all comparisons). Baseline IgE positively correlated with improvement in SALT at week 36 (r > 0.60, p < 0.05). Dupilumab was well-tolerated, with no new safety concerns. These real-world data support the utility of dupilumab to safely treat pediatric AA patients, corroborating the role of Th2 skewing in children with AA and associated atopy, warranting larger clinical trials.

**Supplementary Information:**

The online version contains supplementary material available at 10.1007/s00403-024-03225-4.

## Introduction

Alopecia areata (AA) is a complex, immune-mediated disease characterized by nonscarring hair loss of the scalp and the body that may also involve eyebrows, eyelashes, and beards [1]. AA follows a chronic relapsing and remitting pattern that, in up to 20% of cases, can progress to total scalp hair loss, known as alopecia totalis (AT), or loss of hair on the entire body, known as alopecia universalis (AU) [1–3]. It affects approximately 2% of people during their lifetime, with higher prevalence in children and adolescents [3–5]. About 40% of patients experience disease onset by age 20 [3, 4, 6]. Although two systemic Janus kinase (JAK) inhibitors have been approved for AA, including the JAK1/JAK2 antagonist, baricitinib, for adults [7], and the JAK3/TEC inhibitor, ritlecitinib, for ages 12 and up [8], treatment options for pediatric patients, particularly for those under 12 years of age, are lacking. Further, JAK inhibitors are associated with a black box warning [9, 10], highlighting the need for safe treatments for long term use in the pediatric patient population with AA. This is particularly important, as it has been shown that once JAK inhibitors are stopped, hair shedding is often initiated, with loss of all or almost all hair that has been regrown due to JAK inhibitors, within approximately 3 months [11–13]. In pediatric AA, intralesional triamcinolone (ILTAC) injections and topical corticosteroids remain the first line of treatment, but show limited efficacy in cases with significant hair loss [14]. Additionally, pain and discomfort associated with ILTAC injections and potential scalp indentations make these less practical for children with severe disease [15, 16]. While off label use of oral corticosteroids and other immune suppressants are often given to children with extensive disease, chronic use of these treatments are associated with many side effects [17–19].

Familial or personal atopic diathesis has been identified as the most important risk factor for the development of AA [20–23], with atopic dermatitis (AD) considered the strongest association [20–22, 24–26]. Further, a large population based study showed that each atopic comorbidity increases the risk to develop AA [25]. Concomitant AD has also been shown to increase the risk of more severe AA [23, 25, 27, 28]. IL-13 genetic susceptibility and IL-4 polymorphism, both associated with atopy, were identified in AA [26, 29], and Mendelian randomization analysis also suggests a genetic association between AD and AA [30].The association between AA and atopy is further strengthened by the seasonality of AA [31–33], the positive effect of antihistamines as adjuvants in the treatment of AA [34–36], and findings of allergy-related cellular infiltrates, such as mast cells and eosinophils, in the cellular infiltrates of AA [37–39].

In line with epidemiologic findings, recent serum and scalp profiling of AA patients suggests that, along with Th1 skewing in AA, a Th2 dysregulation also contributes to the pathogenesis of AA, which is more pronounced in patients with associated atopy. The increase in Th2 immune products was also shown to correlate with AA disease severity [37, 40–43] Moreover, recent studies have shown that moderate-to-severe AA presents with systemic immune dysregulation, including increases in markers associated with cardiovascular risk and atherosclerosis, underscoring the need for systemic treatments in severe AA [44]. A hypothesis-driven phase 2a clinical trial in adults with moderate-to-severe AA found that patients with personal or familial atopic background, and/or high IgE levels, who were treated with weekly dupilumab, a monoclonal antibody targeting IL-4 receptor α, had significant improvement in mean Severity of Alopecia Tool (SALT) score compared to placebo at week 24 (primary endpoint), with progressive improvements seen at weeks 48, 60, and 72 [45]. A companion scalp biomarker sub-study of the AA patients treated with dupilumab in this clinical trial showed that the scalp profile of the subset of AA patients with an atopic background was progressively reversed after 24 and 48 weeks of treatment with dupilumab. Moreover, the hair regrowth was associated with attenuation of Type 2 immune pathway genes and with increases in hair keratins at weeks 24 and 48 [42]. Dupilumab is currently FDA-approved for children and adults with AD and with other type 2 inflammatory conditions, such as asthma and eosinophilic esophagitis [46].

Limited data exist on the efficacy of dupilumab in treating children and adolescents with AA, with only single-patient case reports [47–50] and small case series [51, 52] available. While these reports suggest clinical efficacy, they evaluate small numbers of patients, often with short follow up periods, or pediatric patients concurrently treated with other systemic therapy or without active disease. To our knowledge, we are reporting the largest study that evaluates the long-term hair regrowth in 20 pediatric AA patients with concomitant AD treated with dupilumab, identifying significant improvement in AA disease severity.

## Methods

We conducted a retrospective, single-center, observational study at the Icahn School of Medicine at Mount Sinai Department of Dermatology under institutional review board approval (STUDY-22–00500). Adolescents and children, at least 5 years of age and younger than 18 years old, with AA and AD, who qualified for dupilumab (Sanofi and Regeneron Pharmaceuticals, Inc.) due to AD diagnosis and severity, from February 2018 to February 2023, and with at least 6 months of follow-up after treatment initiation, were included. Baseline and follow up photographic images were evaluated blindly by three physicians using SALT scoring. Additional data collected included patient demographics, medical and family history of atopy, dupilumab dosing, previous and concomitant treatments with dupilumab, and IgE levels when available. Adverse events (AE) were also reviewed and further categorized by severity and subsequent management.

To compare AA SALT scores at baseline and at patient follow-up visits, clinical improvement was defined by mean SALT score at follow up visits relative to baseline. Dupilumab 300 mg or 200 mg was given every two weeks, based on patients’ weight and age, after the induction dose (600 mg or 400 mg, respectively), with dosing increased to weekly dupilumab in two adolescent patients. All statistical analysis was performed in R (R-project.org, version 4.3.0). Changes between baseline SALT scores and follow up visit scores were evaluated using unpaired Student’s t-tests. Spearman correlation analysis was conducted to evaluate for associations between baseline clinical characteristics and hair regrowth outcome (SALT).

## Results

### Baseline patient characteristics

A total of 20 pediatric patients were included: 12 females (60%) and 8 males (40%). Mean (± SD) age at dupilumab initiation was 10.8 (± 3.3) years (range 5 to 16 years). Mean (± SD) duration of AA disease was 3.30 (± 3.20) years (range 0.2 to 9 years). Patients in this cohort had a mean (± SD) baseline SALT score of 54.4 (± 37.6) (range 3 to 100), and a majority of patients (75%) had moderate-to-severe AA, defined as SALT > 20 [53], with 7 (35%) of the AA patients presenting with AT/AU. All patients had concomitant AD (100%) and a majority had a family history of atopy (75%) (Tables 1, 2). At baseline prior to initiation of dupilumab, 15 patients (75%) had IgE levels recorded, with an overall mean (± SD) of 1567.7 (± 3270.1) IU/mL (range 2 to 12,760 IU/mL); 8 (40%) had high IgE levels, defined as IgE ≥ 200 IU/mL [45] Mean (± SD) eczema area severity index (EASI) score at baseline was 41.9 (± 8.8) (range: 25–55). Additional baseline patient characteristics are described in Table 1. Over half of the patients in the studied cohort previously tried topical corticosteroids and/or ILTAC with insufficient clinical outcomes (Table 2). Additional atopic comorbidities and prior/concurrent therapies with dupilumab for AA are described in Table 2.

**Table 1:** Patient baseline characteristics

Patient baseline characteristics	(n = 20)
Mean age, years (SD)	10.8 (3.3)
Female sex	12 (60%)
Race	
White	16 (80%)
African American	1 (5%)
Asian	1 (5%)
Other	2 (10%)
Mean SALT (SD)	54.4 (37.6)
Patients with SALT > 75	8 (40%)
Patients with SALT < 75	12 (60%)
Patients with Alopecia Totalis/Universalis	7 (35%)
Patients with personal history of AD	20 (100%)
Mean EASI (range)	41.9 (25–55)
Patients with family history of atopy	15 (75%)
Mean IgE, IU/mL (range) (n = 15)	1567.7 (2—12,760)
Patients with IgE > 200 IU/mL	8 (40%)

*AD* atopic dermatitis, *EASI* eczema area severity index, *SALT* severity of alopecia tool, *SD* standard deviation

**Table 2:** Atopic comorbidities and previous/concurrent AA treatments

Atopic comorbidities and previous/concurrent AA treatments	n (%)
Personal history of atopic conditions	
Atopic dermatitis	20 (100%)
Asthma	6 (30%)
Allergic rhinitis	7 (35%)
Food allergies	4 (20%)
Concurrent treatments for AA	
Intralesional corticosteroids	5 (25%)
Clobetasol shampoo/solution	3 (15%)
Topical minoxidil	1 (5%)
Oral minoxidil	2 (10%)
Topical ruxolitinib	1 (5%)
Previous treatments for AA	
Topical corticosteroids	12 (60%)
Intralesional corticosteroids	11 (55%)
Clobetasol shampoo/solution	6 (30%)
Topical minoxidil	6 (30%)
Oral prednisone	3 (15%)
Oral tofacitinib	1 (5%)

*AA* alopecia areata, *AD* atopic dermatitis, *SALT* severity of alopecia tool

### Dupilumab treatment course and outcome

**Fig. 1:**
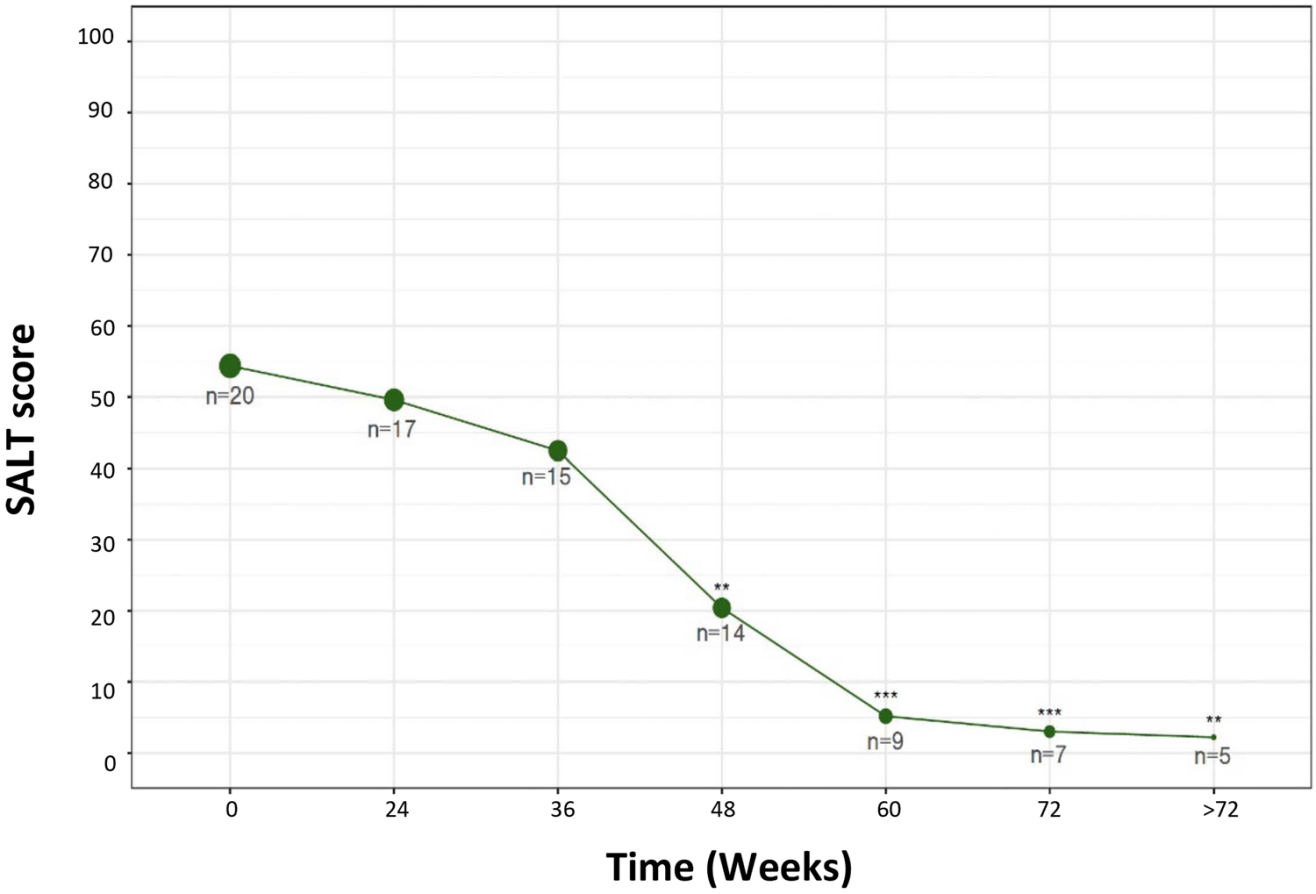
Improvement in Severity of Alopecia Tool (SALT) score over the studied time frame. */**/*** denote significance (p < 0.05/0.01/0.001 respectively) of SALT score at a given time point relative to baseline. The number of patients at each time point is indicated by n

**Fig. 2:**
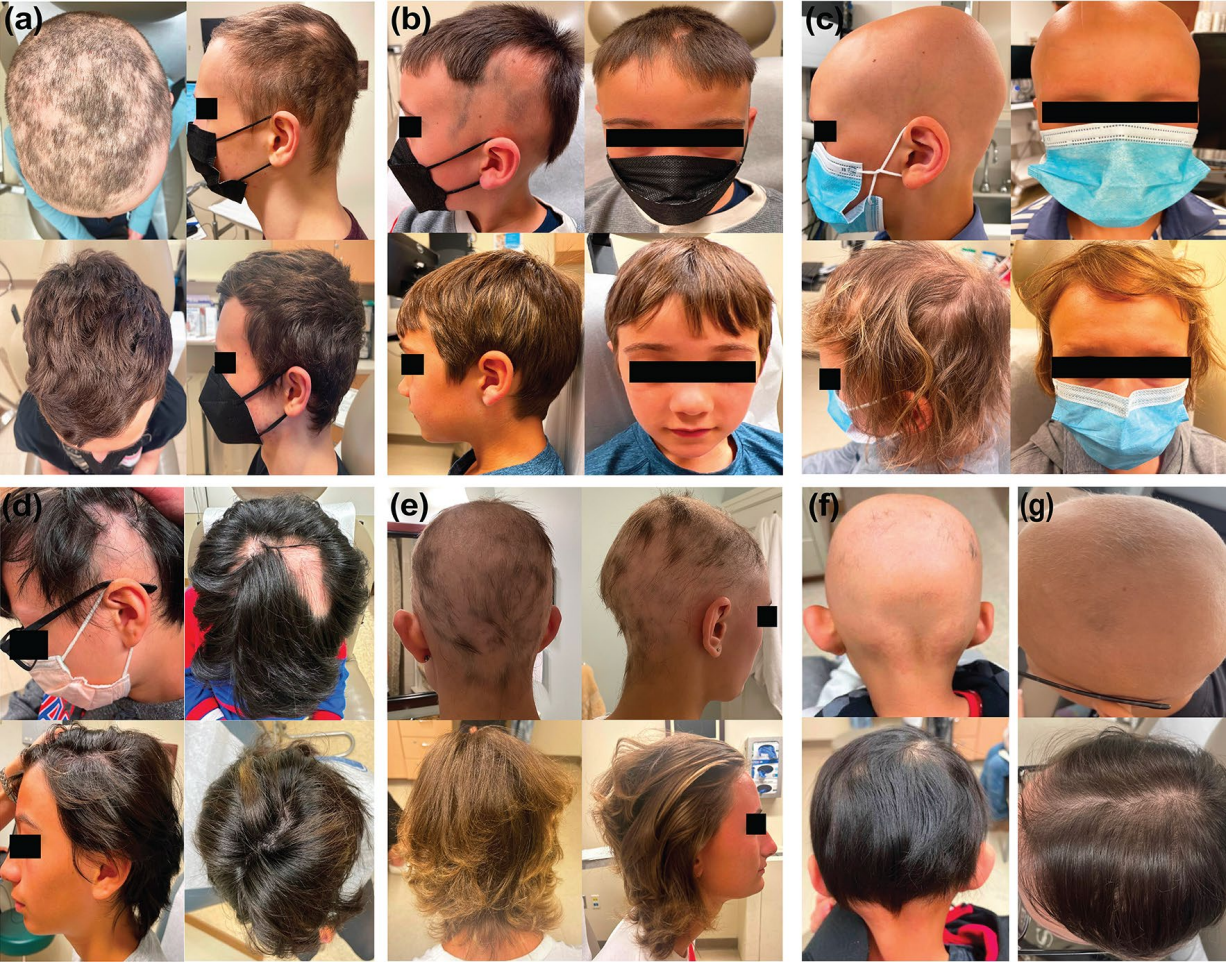
Representative scalp photos of seven patients (**a**–**g**) before and after dupilumab

The mean (± SD) recorded dupilumab treatment duration was 67.6 (± 55.0) weeks (range 24 to 268 weeks). Overall, the patient cohort showed improvement in SALT score over the studied period. Decrease in SALT score was observed as early as week 24, with a statistically significant reduced mean (± SD) SALT score starting at week 48 relative to baseline [SALT: 20.4 (± 35.1) vs 54.4 (± 37.6), respectively; p < 0.01, Fig. 1]. Significant SALT improvement was sustained after week 48, with improvement through week > 72. Mean (± SD) SALT at weeks 60, 72, and > 72 was 5.2 (± 10.6), 3.0 (± 7.9), and 2.2 (± 4.9), respectively (p < 0.01 vs baseline SALT for all comparisons). Among patients with 48 weeks of follow up (n = 14), 86%, 71%, and 57%, achieved SALT50/SALT75/SALT90 improvement, respectively (Table 3, Figs S1-3). Representative pictures of patients’ scalp before and after dupilumab treatment are shown in Fig. 2.

**Table 3:** Proportion of SALT 30/50/75/90 responders at study time points

Proportion of patients achieving SALT30/50/75/90 at week 24	
	All patients (n = 17)
≥ 30% (SALT30)	6 (35%)
≥ 50% (SALT50)	3 (18%)
≥ 75% (SALT75)	2 (12%)
≥ 90% (SALT90)	1 (6%)

Proportion of patients achieving SALT30/50/75/90 at week 36	
	All patients (n = 15)
≥ 30% (SALT30)	10 (67%)
≥ 50% (SALT50)	9 (60%)
≥ 75% (SALT75)	6 (40%)
≥ 90% (SALT90)	3 (20%)

Proportion of patients achieving SALT30/50/75/90 at week 48	
	All patients (n = 14)
≥ 30% (SALT30)	12 (86%)
≥ 50% (SALT50)	12 (86%)
≥ 75% (SALT75)	10 (71%)
≥ 90% (SALT90)	8 (57%)

Proportion of patients achieving SALT30/50/75/90 at week 60	
	All patients (n = 9)
≥ 30% (SALT30)	9 (100%)
≥ 50% (SALT50)	9 (100%)
≥ 75% (SALT75)	7 (78%)
≥ 90% (SALT90)	7 (78%)

*SALT* severity of alopecia tool

When stratifying by the level of IgE (using criteria of ≥ 200 vs < 200 IU/mL), the 8 patients with high IgE levels were found to have responded earlier than the low IgE group to dupilumab treatment (numerical decrease starting at week 24). While patients with low IgE had a slower initial clinical response, they caught up later, ultimately having similar responses by week 60 to patients with high IgE (Fig. S4).

**Fig. 3:**
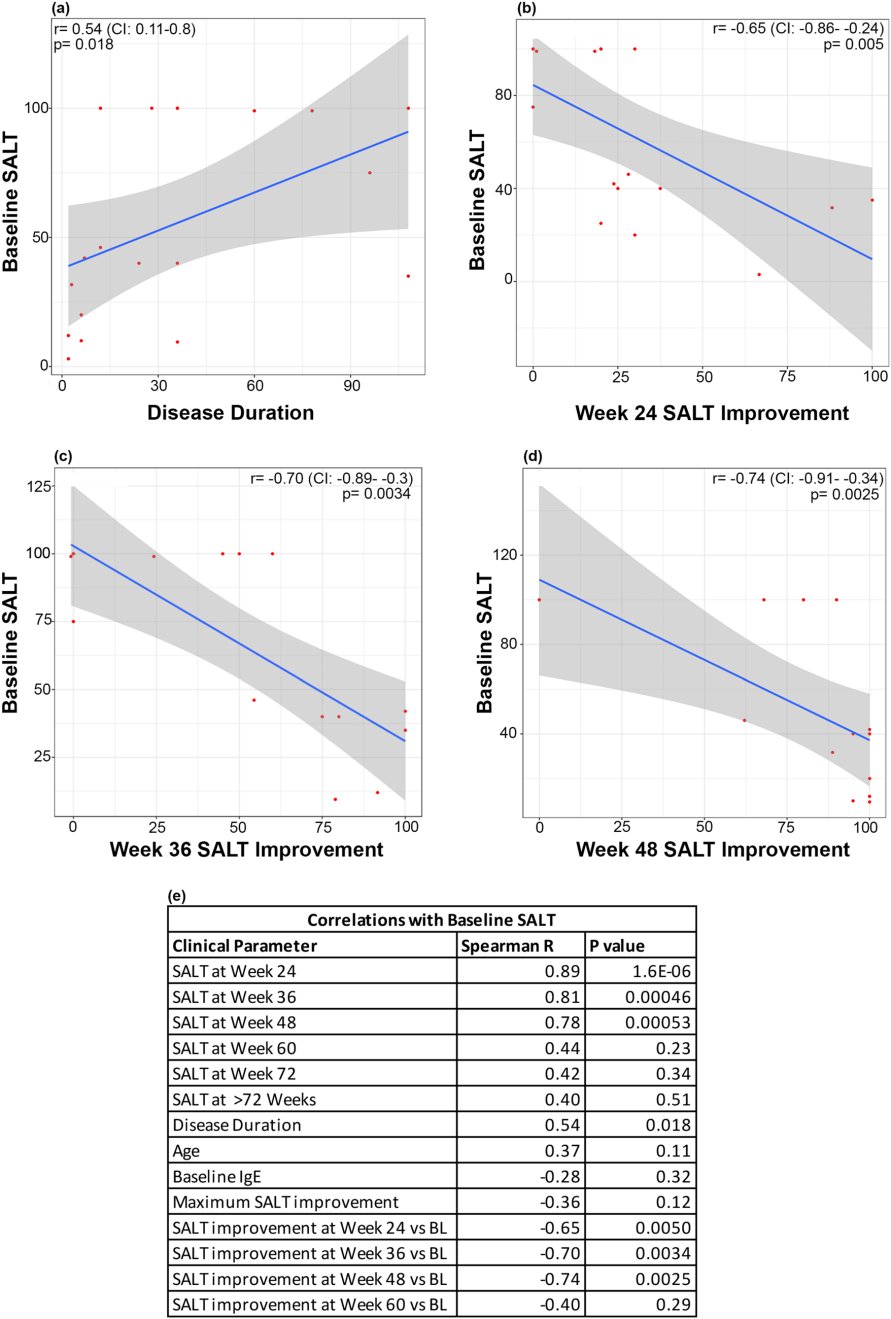
Spearman correlations of baseline SALT with (**a**) disease duration, (**b**–**d**) follow up SALT improvement, and (**e**) summary of baseline SALT correlations

**Fig. 4:**
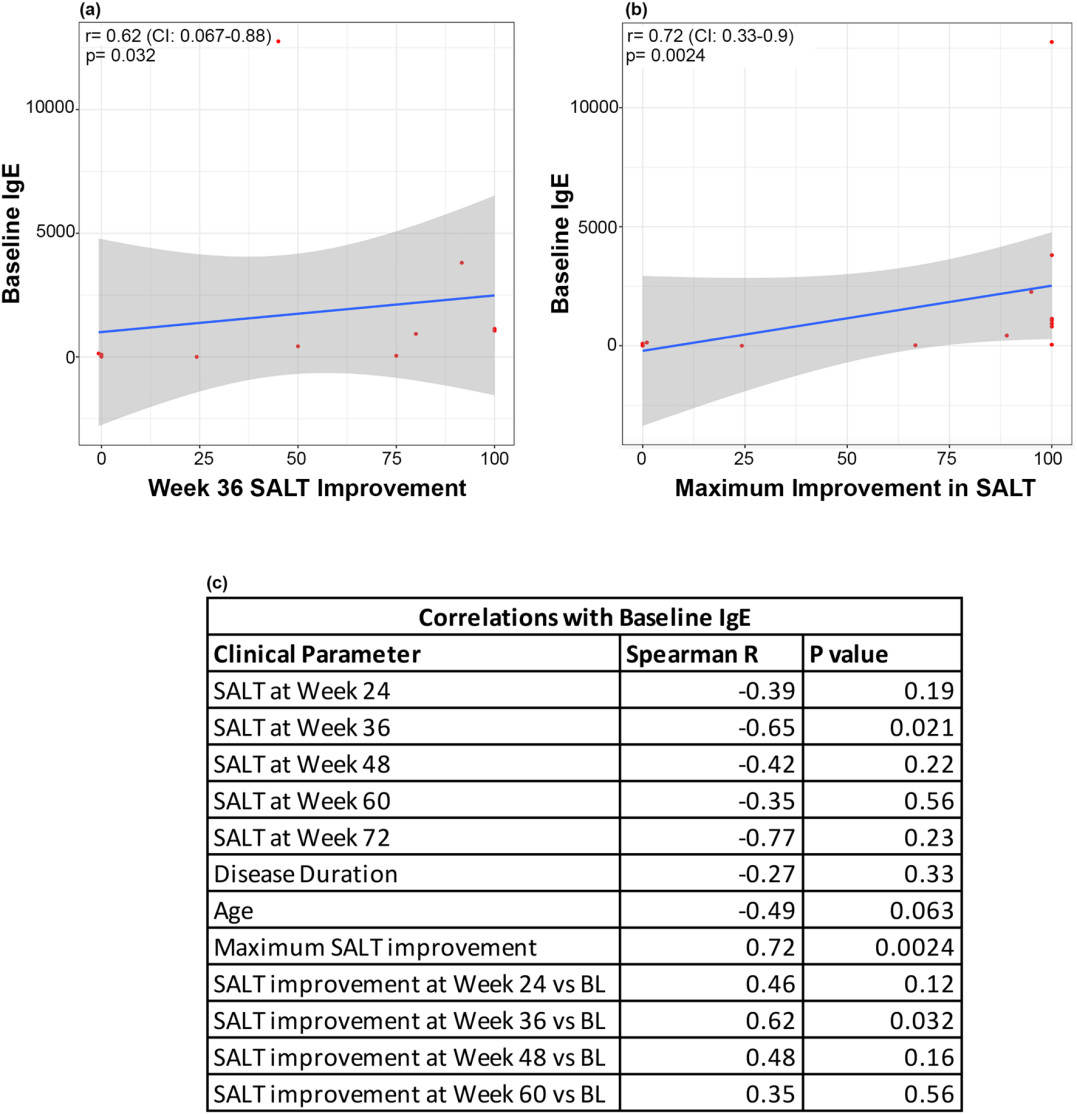
Spearman correlations of baseline IgE with **a** week 36 SALT improvement, **b** maximum SALT improvement, and **c** summary of baseline IgE correlations

In our cohort, baseline SALT was positively correlated with disease duration (r = 0.54; p < 0.05), and negatively correlated with improvement in SALT at weeks 24, 36, and 48 (|r|≥ 0.65; p < 0.01 for all comparisons; Fig. 3A-E). Baseline IgE was positively correlated with improvement in SALT at week 36 and maximum improvement in SALT (r > 0.60; p < 0.05) (Fig. 4A-C). Baseline EASI did not correlate with baseline SALT or improvement in SALT (data not shown).

Among our cohort, 2 patients reported AEs (10%). One patient, with recorded dupilumab treatment duration of five years, experienced joint pain that started approximately three years after starting dupilumab. The patient, who was ANA negative, was referred to pediatric rheumatology, and the joint pain was attributed to a recent growth spurt. There was spontaneous resolution after 1 month without any change to the dupilumab dosing. Another patient developed a new onset facial rash 16 months after starting dupilumab, with rash resolution using emollients after three months. Anaphylaxis, allergic reactions, or other life-threatening AEs were not reported, and overall, the drug was very well tolerated in all patients, with no new safety concerns. There were no reports of conjunctivitis in our pediatric AA and concomitant AD cohort, similar to our clinical trial data from adults with AA [45].

## Discussion

Given the paucity of available effective and safe treatments that can be given long-term for children and adolescent patients with moderate-to-severe AA [14, 54] there is a substantial need to investigate new therapeutic avenues for this large population in need. Herein, we show that dupilumab is well tolerated in pediatric AA with concomitant AD, and results in long-term, progressive and sustained clinical improvement. We found a reduction in SALT already at week 24, and achieving significance by week 48, suggesting that dupilumab in children and adolescents with AA in the setting of atopy necessitates prolonged treatment duration to achieve meaningful clinical benefit. Clinical improvement continued up to a period exceeding 72 weeks. These results in children with AA and concomitant AD mirror the findings of the most recent phase 2a dupilumab trial in adults with AA [45].

In our cohort, patients with longer disease duration had more severe clinical presentation, linking early disease onset to AA severity, underscoring the importance of early disease detection and systemic therapeutic intervention in patients with extensive disease, to enhance the ability to ultimately regrow hair in this population [55]. In line with previous studies in adult AA cohorts [45, 56], we also found that baseline SALT negatively correlated with clinical improvement at weeks 24, 36, and 48.

Therapies currently used in children with AA have limited clinical efficacy and/or questionable safety profiles. Inadequate treatment options for pediatric patients may exacerbate the pre-existing psychosocial and emotional burden on this vulnerable patient population [57]. AA in children takes an emotional toll on the patient as well as on the entire family [58, 59], with some reports showing that AA disease severity directly correlates with impact on patient and family quality of life [59, 60]. Earlier onset AA is associated with more severe clinical presentation and disease progression [2, 61]. In turn, early detection and intervention with effective and safe therapeutics for long term use are imperative, especially in more severe pediatric AA patients [14]. The oral dual JAK3/TEC family kinase inhibitor, ritlecitinib, has recently become the first FDA approved treatment for adolescents with AA [62], but safety concerns remain, in particular with long term use in pediatric AA populations [63, 64], as it is associated with a black box warning [10]. In contrast, dupilumab safety profile has been well studied in multiple multi-year longitudinal studies, does not necessitate routine blood monitoring, and has an acceptable safety profile in children as young as 6 months old [65, 66]. In our present study, no serious AEs were associated with dupilumab use. Only two patients experienced mild AEs, joint pain and transient facial redness, which has been previously reported in AD patients treated with dupilumab [67–71]. Of note, similar to our clinical trial data from adults with AA [45], no eye manifestations were seen in our children with AA and AD treated with dupilumab. Future studies should clarify if indeed the eye manifestations of dupilumab are only seen in the context of AD alone, as these were also not seen in asthma [72, 73] or other indications of dupilumab [74, 75].

Th2 targeting with dupilumab in pediatric AA patients with concomitant atopy may not only expand treatment options, but may also enrich our understanding of the pathogenic role of Th2 in the entire AA population, and in particular within the subset of patients that have atopy [37, 40–43, 76, 77] Further, while JAK inhibitors show promise and have been approved in adults and adolescents with AA, they have broad mechanisms of inhibition and target multiple cytokine pathways, precluding the identification of the pathogenic contribution of specific immune pathways to AA pathogenesis [78]. The increases in key Th2 biomarkers (i.e. IL13, CCL17, CCL18, CCL22, CCL26) in AA scalp, particularly in atopic individuals [37, 40, 41], and systemic immune activation of this pathway that correlates with AA severity [44], together with the efficacy of dupilumab in the clinical trial in adults with AA and atopy [45], highlight the potential applicability of Th2 targeting with dupilumab in children with AA and atopy. Further analyses may also elucidate which biomarkers can predict the optimal AA patient candidates for dupilumab use. Dupilumab may thus provide a targeted and safe treatment for long term use [65, 79, 80] that can reduce both local and systemic inflammation, as shown in AD [81, 82], while inducing and maintaining hair regrowth.

Our study includes some limitations, such as the retrospective study design, modest sample size, and lack of a comparator group. Nevertheless, this is the largest reported cohort of pediatric AA patients treated with dupilumab over an extended follow-up period. Future, larger studies, including randomized placebo-controlled clinical trials, are warranted in children and adolescents with concomitant AA and atopy, to further evaluate the benefit of dupilumab for the long-term management of AA in pediatric patients with associated personal and familial atopic comorbidities.

## Supplementary Information

Below is the link to the electronic supplementary material.

Supplementary file1 (PDF 619 KB)

Supplementary file2 (PDF 624 KB)

Supplementary file3 (PDF 622 KB)

Supplementary file4 (PDF 230 KB)

## Data Availability

The data that support the findings of this study are available from the corresponding author upon reasonable request.

## Abbreviations

AA: Alopecia areata
AD: Atopic dermatitis
AE: Adverse event
AT: Alopecia totalis
AU: Alopecia universalis
FDA: Food and drug administration
ILTAC: Intralesional triamcinolone
JAK: Janus kinase
SALT: Severity of alopecia tool
