# Supplementary material for: Dupilumab induces hair regrowth in pediatric alopecia areata: a real-world, single-center observational study
Source: Arch Dermatol Res. 2024 Jul 23;316(7):487. doi: 10.1007/s00403-024-03225-4 (PMC11266226; doi:10.1007/s00403-024-03225-4)
Supplement: Supplementary file 3 — Supplementary file3 (PDF 622 KB) [file 403_2024_3225_MOESM3_ESM.pdf]

Supplementary information for “Dupilumab induces hair regrowth in pediatric alopecia areata: A real-world, single-center observational study”.

Journal: Archives of Dermatological Research

Author Names: Eden David BA[1]*, Neda Shokrian BA[1,2]*, Ester Del Duca MD, PhD[1], Marguerite Meariman MD[1], Jacob Glickman MD[1], Sabrina Ghalili MD[1], Seungyeon Jung[1,3], Benjamin Ungar MD[1], Emma Guttman-Yassky MD, PhD[1]
*these authors have contributed equally and are designated to have co-first authorship

Author affiliations:
[1]Department of Dermatology, and Laboratory of Inflammatory Skin Diseases, Icahn School of Medicine at Mount Sinai, New York City, New York, USA
[2]Albert Einstein College of Medicine, New York, NY, USA
[3]School of Medicine, Vanderbilt University, Nashville, TN, USA

Corresponding author email: emma.guttman@mountsinai.org

Figure S3

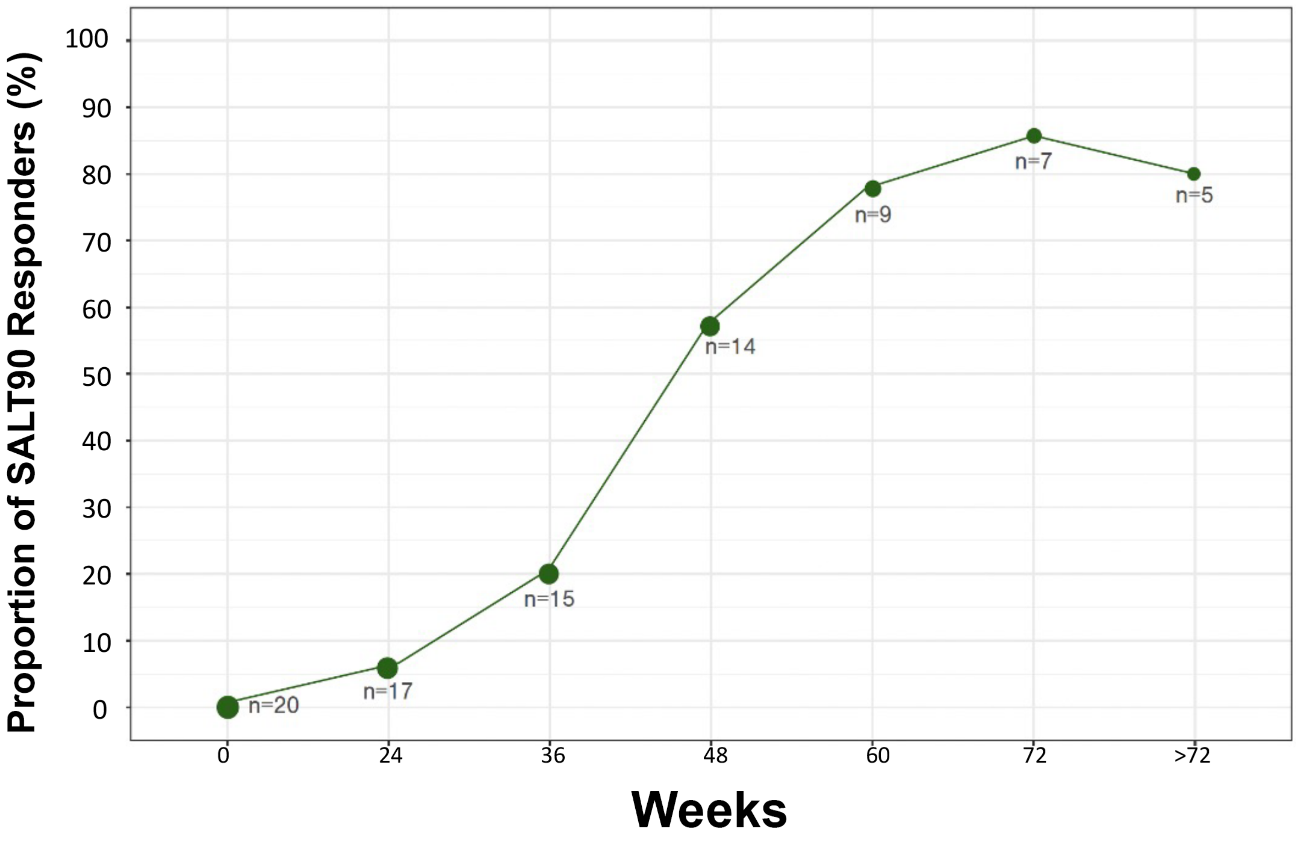
Proportion of SALT90 Responders (%)
100
90
80
70
60
50
40
30
20
10
0
n=20
n=17
n=15
n=14
n=9
n=7
n=5
0
24
36
48
60
72
>72
Weeks
